# How an Environment of Stress and Social Risk Shapes Student Engagement With Social Media as Potential Digital Learning Platforms: Qualitative Study

**DOI:** 10.2196/10069

**Published:** 2018-07-13

**Authors:** Becky Hartnup, Lin Dong, Andreas Benedikt Eisingerich

**Affiliations:** ^1^ Imperial College Business School Imperial College London London United Kingdom

**Keywords:** social media, online learning, digital engagement, stress, social risk, digital platforms, education, university adjustment

## Abstract

**Background:**

Social media has been increasingly used as a learning tool in medical education. Specifically, when joining university, students often go through a phase of adjustment, and they need to cope with various challenges such as leaving their families and friends and trying to fit into a new environment. Research has shown that social media helps students to connect with old friends and to establish new relationships. However, managing friendships on social media might intertwine with the new learning environment that shapes students’ online behaviors. Especially, when students perceive high levels of social risks when using social media, they may struggle to take advantage of the benefits that social media can provide for learning.

**Objective:**

This study aimed to develop a model that explores the drivers and inhibitors of student engagement with social media during their university adjustment phase.

**Methods:**

We used a qualitative method by interviewing 78 undergraduate students studying medical courses at UK research-focused universities. In addition, we interviewed 6 digital technology experts to provide additional insights into students’ learning behaviors on social media.

**Results:**

Students’ changing relationships and new academic environment in the university adjustment phase led to various factors that affected their social media engagement. The main drivers of social media engagement were maintaining existing relationships, building new relationships, and seeking academic support. Simultaneously, critical factors that inhibited the use of social media for learning emerged, namely, collapsed online identity, uncertain group norms, the desire to present an ideal self, and academic competition. These inhibitors led to student stress when managing their social media accounts, discouraged them from actively engaging on social media, and prevented the full exploitation of social media as an effective learning tool.

**Conclusions:**

This study identified important drivers and inhibitors for students to engage with social media platforms as learning tools. Although social media supported students to manage their relationships and support their learning, the interaction of critical factors, such as collapsed online identity, uncertain group norms, the desire to present an ideal self, and academic competition, caused psychological stress and impeded student engagement. Future research should explore how these inhibitors can be removed to reduce students’ stress and to increase the use of social media for learning. More specifically, such insights will allow students to take full advantage of being connected, thus facilitating a richer learning experience during their university life.

## Introduction

### Background

Medical education is about more than developing necessary technical knowledge and skills; interpretation and communication skills are also required. Doctors need to be able to understand people, to empathize, and to break bad news. As students, and then as practitioners, those working in medicine must learn to deal with ambiguity and associated risks. This factor can cause student frustration and anxiety and, potentially, burnout.

As a medical undergraduate student noted in an interview with us, “Medical students are often anxious, questioning if they have made a right decision. Should they persist or jump?” The medical student further noted that:

We set up a precourse Facebook group for students. It helps us to bond. That is important because medicine needs to be collaborative. Why is collaboration so important? Medical practitioners need to learn about collaboration, teamwork, acknowledgement of uncertainty, knowing when to be curious and when to be brave, understanding the nature of risk.

However, in the interview, the medical student also noted that:

Additional pressure on medical students exists with what they post on social media because of fitness to practice. If they say something inadvisable on social media, it can be held against them in their career. As a result there is a swing from more public forums to Snapchat where things do not persist permanently. Managing multiple images is more difficult for medical students.

The increasing ubiquity of social media use in medical education has transformed how students learn and interact with their peers at school and beyond [1,2]. For example, universities encourage online collaborative learning. Students participate in online microcommunities to facilitate academic discussion, especially when they enter a new environment that requires fast adaptation [3,4]. For medical students, using social media to seek academic support is particularly pervasive.

Medicine is one of the most information-rich professions, where scientific progress is rapid and scientific breakthroughs happen almost on a daily basis [5]. Despite the wide recognition for social media’s potential to facilitate knowledge sharing and encourage discussion, we still lack understanding of the key factors that motivate or inhibit students’ engagement with social media as an effective tool for learning.

Thus, we aimed to explore two important questions: what role does social media play in medical students’ personal lives and learning experience in their phase of university adjustment? How does such experience affect their engagement with social media?

University adjustment, also recognized as emerging adulthood, is a distinct period in students’ lives [6] (pg 469). It refers to the transition phase to university. More specifically, students move away from their long-term social relationships, reestablish themselves with new social groups [7], and explore their roles and identities in new and unfamiliar environments. In addition to this, they need to cope with academic pressures. While adults may have a better capability to adjust to various life transformations, research into this earlier life stage and the challenges of transition has highlighted the potential negative impacts on mental health linked to failure to adjust, which, in turn, can affect students’ academic performance [8]. In contrast, successful adjustment has been noted to facilitate better academic results and, therefore, better life outcomes [9].

Notably, on average, medical students experience a higher level of depression and anxiety [10,11]. A combination of academic training, clinical visits, internal examinations, and external licensing examinations can be stressful for medical university students [12,13]. They may need to visit patients, be responsible for taking care of them, and prepare themselves to perform irreversible, high-risk treatments in the future [14]. These pressures add more challenges to medical students’ university life and to going through the phase of university adjustment.

Research has suggested that social media might facilitate students’ successful university adjustment, well-being, and learning outcomes [15]. To cope with pressures in the new environment, students use social media to communicate with friends, to seek academic support, and to relax. However, these online activities are visible and might also create social risks [16-18]. In the offline environment, people manage their identities and the perceptions that others have of them in social interactions using social monitoring to fit different versions of themselves to different social groups and conversations. This method is untenable online because social media brings different social groups into one common place [19]. People now have pressures to present a single online identity to multiple offline audiences [20]. For university students, these audiences could be old friends, new friends, and academic peers.

Except for complex audience groups, on social media, users are able to create an idealized version of themselves [21,22] by, for example, selectively posting photos and sharing articles. It helps to build their desired personal images among the audience to increase self-acceptance [23]. For example, students may use Instagram, a visual platform, to share carefully edited photos of themselves and evaluate the popularity of these posts [24]. These concerns complicate the process when students try to use social media to manage their social relationships and improve their academic performance.

### Objective

Critical gaps remain in our understanding of what drives students to actively engage with social media and the barriers that impede such engagement in the phase of university adjustment. The lack of understanding prevents us from maximizing the benefits that social media can provide for students, such as creating new opportunities for learning and enhancing learning efficiency.

Thus, this study, we aimed to (1) investigate the drivers and inhibitors of social media engagement among first-year undergraduate students in medical schools, and (2) based on our findings, develop a framework for understanding students’ behavioral patterns on social media platforms and their effects.

## Methods

### Overview

Because of the limited understanding of medical students’ online behaviors in university adjustment, we conducted a qualitative study, as it suited the exploratory nature of the research [25]. We carried out 78 in-depth interviews with undergraduates in medical schools about their online behaviors and 6 interviews with digital technology industry experts. With the permission of our interviewees, we recorded and transcribed all the student and industry expert interviews. We used thematic analysis to identify the emerging themes and patterns [24]. We obtained ethics approval for this study from the Imperial College London, London, UK.

### Participant Recruitment

#### In-Depth Interviews With Students

We conducted all interviews face-to-face with 78 undergraduate students studying academic medical courses at universities in the United Kingdom. Interviews took place at the end of the summer holiday and during the fall term of 2017. Because social media use is a personal topic to discuss, we adopted an informal tone that suited the students in order to make them feel comfortable and be willing to reveal their true self [24]. Some students also showed us their social media pages to demonstrate their views. Students were informed of the broad scope ahead of the interviews and reassured regarding confidentiality of their answers.

We recruited interviewees through posters on university campuses. The main criteria were availability for a face-to-face interview and willingness to talk about personal social media behaviors. The initial interviews were relatively open and led by the interviewees, around the broad themes of social media use and university adjustment. As the interviews progressed and themes began to emerge, the interviews became semistructured, but with room for interviewees to open their discussion onto the issues that they felt were more relevant in the context of social media use and online learning.

#### Interviews With Industry Experts

We carried out interviews face-to-face and by telephone with 6 digital technology experts. We employed convenience sampling among interviewees that we approached through our personal network. More specifically, these experts had worked in a digital learning environment for several years. Some of them helped build online courses at a research-led university in the United Kingdom. Others had worked in a digital learning environment (developing or managing online courses) internationally. The primary purpose was to obtain additional insights into students’ engagement with social media in the context of learning and additional views on students’ behavioral patterns on social media.

### Data Analysis

One author (BH) used thematic analysis to identify the drivers and inhibitors of social media engagement. First, we separated the data into meaningful fragments about how, why, and when students used social media. We labeled these fragments using descriptive codes.

Then we looked for the relationships among these codes and aggregated them into themes such as maintaining existing relationships and building new relationships. We sought to combine these themes and develop a simpler and parsimonious framework that demonstrates drivers and inhibitors of social media use, the elicited stress, and behavioral outcomes.

## Results

### Summary of Findings

All interviewees were living away from home and were between the ages of 19 and 22 years. The interviewees were ethnically diverse, and 49 of 78 (63%) interviewees were female.

Combining our student interviews with the experts’ observations, we found that maintaining existing relationships, building new relationships, and seeking academic support drove students to engage with social media and use it for learning. The key inhibitors that emerged from our data were (1) collapsed online identity, (2) unclear and even conflicting norms, (3) the desire to present an ideal self, and (4) perceived academic competition within their social groups. The findings also highlight that students engaged with various social media platforms in different ways. In the following section, we elaborate on the key drivers and inhibitors of social media engagement in greater detail.

### Drivers of Social Media Engagement

**Figure 1:**
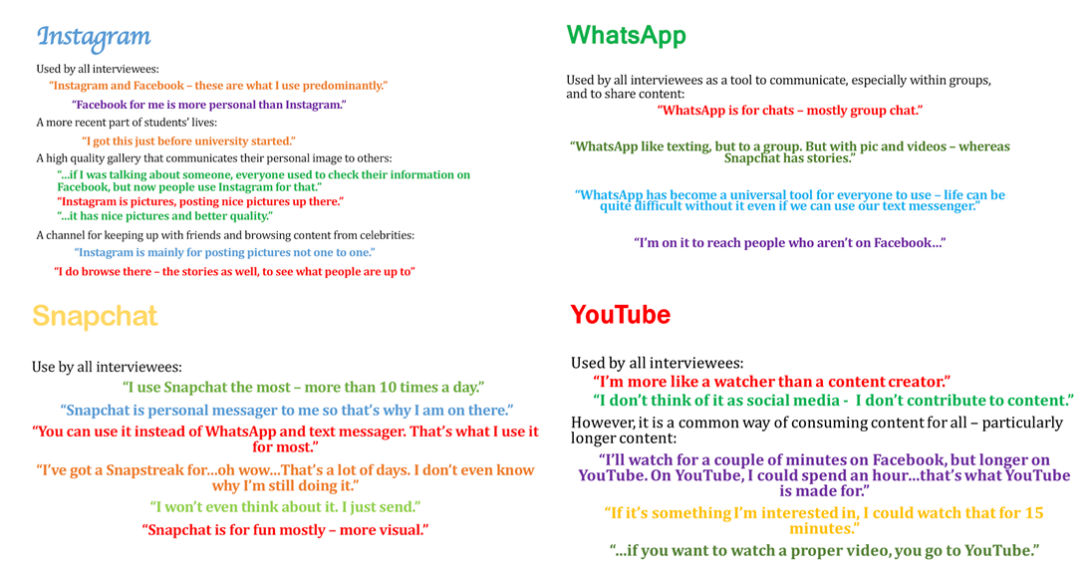
Social media use by medical undergraduate students.

First, our findings showed that social media was critical for *maintaining existing relationships*. Students engaged with social media because they wanted to stay in touch with their friends at home after entering university by checking the content created and shared by their friends from home. Figure 1 shows sample quotes posted to Instagram and Snapchat, including quotes relating to maintaining existing relationships. As Table 1 shows, numerous students mentioned social media’s role in keeping relationships they had prior to joining university. The effort to maintain old friendships helped students to reactivate them easily when they returned home. In addition, social media provided social support for students when they faced difficulties in university, as the student quotes in Table 2 illustrate.

Second, at universities, students *built new relationships*. Such new relationships formed a significant part of students’ experience at university and beyond, as this experience may shape their future social network and influence potential opportunities related to employment after graduation. As a result, students usually made some effort to explore more information about their new contacts. Social media offered a shortcut to gain deeper insights into new contacts and people they had never met before.

**Table 1:** Samples of student interview quotes: social media for maintaining existing relationships.

Interviewee number	Sample quote
8	Instagram allows me to stay in touch with my old friends from home. It makes me feel I am not alone at uni. It’s just nice.
24	Sometimes the posts on Instagram make me feel connected to home and my friends there. We share clips. I tell them about my uni life and they share interesting articles and clips with me. I even use it for my assignments.
47	It is how I keep in touch with people. Without Facebook I wouldn’t be friends with those people.
50	See what pictures they’re posting, what they’re up to. Gives me an idea of what’s going on with them. Otherwise, I wouldn’t know what’s going on—I’d go back home once a year and listen to their updates from the whole year. But I can listen to their updates every day.
2	I wouldn’t want to miss my friends from home. Use Snapchat to share and send stuff. Makes me happy and helps me with stress.

**Table 2:** Samples of student interview quotes: social media for social support.

Interviewee number	Sample quote
11	I can’t ever imagine what it would be like to have gone off to different universities and not have the things we have—I wouldn’t be friends with half the people I am now. You can send someone a Snapchat once a week and keep that friendship as strong as when I left. It was comforting. We had a shared WhatsApp group and we all knew where everyone was going, so it was like “What’s yours like?” It was like a safety net.
69	Looking at all these new profiles can be overwhelming. When I see a familiar face on Instagram or Snapchat it puts a smile on my face.
17	For me it’s incredibly important to keep my friendships. I don’t want to lose my friends from home. Otherwise they will ask “hello stranger, where have you been?” WhatsApp chats keep us connected.
30	I would feel very lonely with my old friends. They know me so well. I know them. It’s just different with new people. I connect with my friends on a regular basis. They are on my WhatsApp. We also use Instagram. Without them I don’t think I would have had the same experience at university.

**Table 3:** Samples of student interview quotes: social media for seeking academic support.

Interviewee number	Sample quote
54	A couple of friends have asked me about how I do a problem; sometimes without telling the answer I show them the workings so it helps with actually...helping people when they are not even there. If they are all the way over in South Kensington I can just help them and tell them how to do a problem without even seeing them. And it feels good to do that...Facebook has enabled me to help others and also to receive help when I need it.
4	Organizing all these group meetings can be a bloody nightmare. Without WhatsApp it would be impossible. I honestly have no idea how students in previous generations managed without WhatsApp.
18	We connect via Snapchat all the time. We even use it during lectures and classes. Don’t always feel comfortable asking the lecturer. Then I ask my peers on WhatsApp if they understand something. Or we decide who to ask the lecturer during the class. Makes learning easier.
46	Group assignments, feedback, and topics are all discussed on WhatsApp. Instagram and Snapchat are more for entertainment but that’s also important sometimes. WhatsApp is used for group discussions and preparations more than any other app and definitely more than the university app offered.
9	For each group exercise we form a chat group on WhatsApp. Sometimes we also share on Instagram.
27	Even one of our lecturers connected with students on WhatsApp. Some students said this was not appropriate, but others liked it. Of course only academic content should be shared and discussed maybe.

According to a student interviewee:

I would know a person more if I have looked at their profile and understand what kind of things they are doing or what kind of status they like or what kind of pages they like.

Interviewee #3

It should be noted that social media not only was an independent platform that students used to build direct online relationships, but also integrated their online and offline social interactions. As a student shared with us:

For example, for nights out people will say if they’re interested or if they’re willing to go so I’ll look at who is going, who is interested and make my decision as to whether I should go.

Interviewee #20

Third, students saw social media as an important tool to *seek academic support*. It provided a convenient platform to create and maintain social ties for academic purposes, such as group work for assignments and examinations. For example, Table 3 lists some examples of how students thought about social media in terms of academic support.

### Inhibitors of Social Media Engagement

Interestingly, social media assisted students to deal with challenges related to university adjustment; however, the interaction of relationship management and academic activities triggered multiple concerns. First, maintaining existing friendships and building new ones led to *collapsed online identity*. Students needed to manage their identity across multiple audience groups. The types of audiences determined the level of self-revelation. Students directly linked this concern to being more cautious when posting on social media (see Table 4).

Second, students were *uncertain about social norms* when establishing new networks. Students’ online activities could be evaluated by various audiences, depending on social and relational contexts. During the university transition period, students’ networks expand and become more diverse, with the potential for a clash of social norms. Most of the social media activities are documented and visible, so students tended to be more conservative about their online activities (eg, see Figure 1). One student shared with us about his experience that:

There have been one or two occasions after two weeks when I haven’t posted anything...I’ve been seeing other people’s videos, or I’ve been liking people’s pictures on Instagram, but I haven’t contributed on my profile. I haven’t contributed on Instagram. Lots of other people have been commenting on how they’ve been spending their time and I’ve not been doing that. I’ve had a couple of occasions when I’ve been thinking why I haven’t posted stuff...Probably it’s because I’ve forgotten. Since more recently I’ve been more into what I’ve been posting I will have that sense of something is missing. This awkward feeling “should I possibly post something or should I not?” If you’d asked me that question a year ago when I was at school I honestly would not have cared.

Interviewee #26

The uncertainty about social norms encouraged students to observe others’ behaviors in order to identify the appropriate and acceptable online behaviors. As a student told us,

Before I post anything on WhatsApp in the discussion groups, I check what others have to say. I do not want to be the first one to post and share.

Interviewee #76

Third, students saw the profile on social media as a proxy for self and wanted to *present an ideal self*. As students actively reviewed others’ profiles, they became aware that they were also being judged by others, which may bring social risks if they do not manage the account appropriately (Table 5).

**Table 4:** Samples of student interview quotes: collapsed online identity as an inhibitor to using social media for learning.

Interviewee number	Sample quote
65	The people who know me in the university versus the people who know me back in [my home country]—it’s a different person. If I was travelling I wouldn’t mind posting, but a personal newsfeed—I wouldn’t put something on there.
12	I do not share everything with everyone. I do not feel comfortable asking for help sometimes. It would make me look stupid in front of some people. I would not like that.
21	I don’t want everyone to see what I wear every day or where I hang out. When I share a picture of myself on Instagram, it’s out there. Snapchat is easier. Few people can see it and then it’s gone.
50	Some may judge you for doing things. I don’t like that. On social media everything gets judged by everyone all the time. That’s social media for you nowadays.
56	On Facebook you don’t know who can see your profile. People share information freely. I am uncomfortable disclosing my status to everyone in my learning group. I rather not share at all. I don’t want all of my study group members to see my pictures and who I am with. I’d rather keep things separate.

**Table 5:** Samples of student interview quotes: social media and presenting an ideal self.

Interviewee number	Sample quote
10	I’ve made an active change because my Instagram used to be trashy. Say it was a friend’s birthday or we were drunk. It wouldn’t be good quality and the captions would be inside-jokes. And then I realized, when I went to university, if I was talking about someone, everyone used to pull them up on Facebook, but now people pull them up on Instagram. It would be shameful. So now it’s nice pictures and better quality.
40	If I’m choosing a profile photo – I’ll ask other people’s opinion. I want to look good, I want it to look nice.
12	It can take me forever to take a selfie. I naturally want to look good in the pictures I share with people that just met me. With my old friends it is not a big issue. They know me but for new people I want to be at my best. I go shopping for new T-shirts, bras, put on makeup and take at least 20 different pictures.
13	There’s a lot of pressure to look at your best. It’s a real problem for many people. Cyberbullying and body judgements are a constant. I feel uncomfortable posting a picture on Instagram and others not liking it. Nobody wants to look like a fool. I am worried to share questions on Facebook and WhatsApp. I know other people must think I am not smart or ask stupid questions.
33	Instagram makes me feel I am not leading a great life. Everyone else looks so fab in their pictures. Big smiles and all that. Everyone is running marathons and looking hot, playing cool, studying hard. I feel the pressure to do the same. It’s not really me but I feel I have to do it.
9	People comment on others saying ‘this is a dumb point’ or ‘you are not smart enough to be here’. It’s mean. Then you have people posting all these super-genius things. People use social media to look smarter than they are. Definitely I try to use WhatsApp that way. Everyone does.

When managing their posts and comments, students also wanted to attain endorsement from their peers through “likes.” This is important to their self-esteem because likes signal a student’s popularity and status among their peers. According to one of our interviewees,

...if I change a profile picture I’m not anticipating thousands of likes, but some would be nice. If its no-one I would be quite disappointed.

Interviewee #45

The desire to idealize the self on social media made visible behaviors, such as posting and sharing, became high-risk activities that required cognitive effort. It also shaped students’ perceptions of offline reality. For example, a student shared with us that

...coming into university, I do feel that how you perceive yourself online has a great impact on what reality is like.

Interviewee #59

Thus, the relationship between online identity and self was more complex than a straightforward self-presentation on a digital profile. Any visible engagement on social media contributed to external perceptions of an individual and to the way in which an individual saw and felt about themselves.

Fourth, because of the visibility of social media activities, posting related academic content might elicit a sense of *academic competition*. Even though academic communication plays a central role in students’ daily lives, sharing relevant information online might be sensitive. Students tended to be cautious about whether the learning content should be shared with their peers. According to our interviewees,

If it is very close friends, we will share everything...but there is a line between being friends and being in competition. I wouldn’t want to share everything.

Interviewee #7

No way, I am not sharing my studies with peers. They are my competitors. If they find out what I read, they will read it too. I do not share my learning material on social media at all. Once it’s on social media, it is everywhere and my competitive advantage is gone.

Interviewee #63

**Figure 2:**
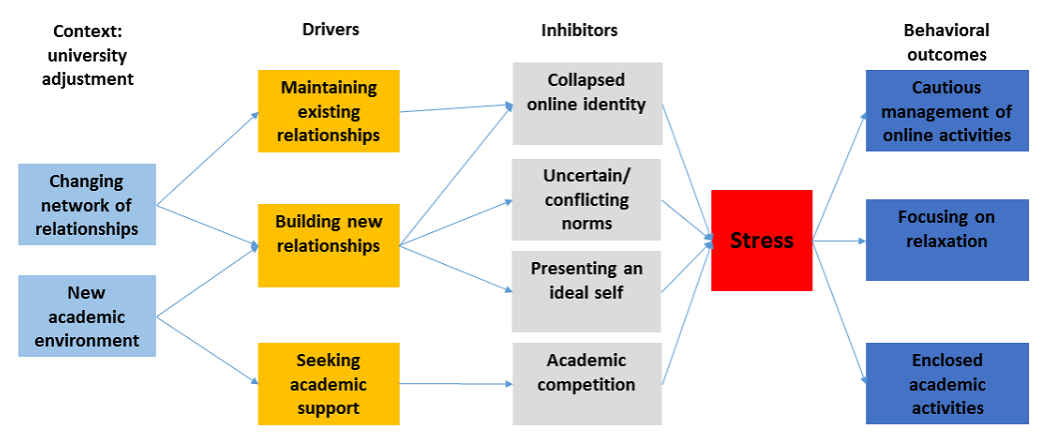
Social media engagement in university adjustment: framework of drivers and inhibitors.

### Stress

The interaction among drivers and inhibitors of using social media mentioned above consequently created psychological stress for students. First, students needed to manage their online identities to appeal to new friends. They edited their posts and managed other online activities carefully. Students were particularly concerned about the norms related to how to behave in universities in order to fit in.

Second, students faced the challenges of establishing an online persona on social media for different audiences, including old friends from home and new friends from university.

Third, the pressure from academic studies also acted as both motivation and inhibition for students. They used social media to work with their peers. However, this process led to a sense of competition in which students may put extra efforts into evaluating whether the learning content should be shared.

### Behavioral Outcomes

Three behavioral patterns emerged in response to stress. First, students became more cautious about their online activities to manage their identity and guard against the offline impacts. They may have sought private solutions to support their educational attainments, such as searching for online content or contacting their close friends for discussion. Second, they passively consumed content with positive valence for relaxation. Third, students relied on social media such as YouTube (see Figure 1) to reduce their level of stress. As shared by our interviewees, using social media to relax after a busy and stressful day at university played a big part in their university life.

At the end of a long day YouTube is my best friend. It is super relaxing.

Interviewee #29

I use YouTube to learn and watch new things. It also motivates me and keeps me going.

Interviewee #44

YouTube offers great lecture series by different universities. Often the recording is of better quality than the recordings of my lectures at uni.

Interviewee #36

It’s not like I don’t want to study, it’s just that I am tired at the end of the day. I want to put on my comfy clothes and watch some funny clips on YouTube.

Interviewee #25

Experts have also been aware of the challenges of university adjustment for students and how students engage with different social media as potential learning platforms. Experts affirmed that students at the phase of university adjustment are difficult to engage on social media. Within private groups, where students are not observed, they communicate freely. Even though both experts and students acknowledge the potential for social media to deliver a transformed learning experience, the outcome highly depends on the engagement behaviors of students. To offer more learning support on social media, relevant stakeholders such as universities and publishers need to eliminate students’ stress and encourage them to actively engage on social media.

On the basis of our interview findings, we developed a framework that highlights the driving factors and inhibiting factors that can help us to understand students’ social media engagement behaviors stemming from relationship management and fitting into the academic environment in university adjustment (see Figure 2).

## Discussion

### Principal Findings

That students’ social media activities are heavily influenced by an environment of stress is not a particularly new finding. Students during university adjustment may face consistent volatility of their lives, including regularly changing living arrangements, social and romantic fluidity, and flexible courses where they meet new classmates with each module. Wohn and LaRose [8] described this as a period “fraught with both psychological and academic stress” (pg 158). Adding to our understanding of social media use among students, this study clarified how relationship management in a new academic environment shapes their social media engagement behaviors for learning.

This study’s findings extend those of previous studies, which showed that social media empowers students to manage their existing and new relationships, and to support their learning [3,10,11]. In the phase of university adjustment, however, the interaction among these factors impedes students’ engagement with social media. This study can inform educators and publishers and help them to better understand the challenges of engaging students online when promoting social media as a learning tool.

In line with prior work, this study indicates that social interaction and social support are the most common motivations to use social media [22,26,27], particularly on Facebook [28]. Specifically, social media facilitates friendship establishment and maintenance because it allows communication without time and distance limitation [21]. For university students, social media, such as Instagram, Facebook, and WhatsApp, enables them to stay close to their home and school friends [9]. This may help reduce the negative effects of friendsickness (defined by Paul and Brier as the “preoccupation with and concern for the loss of or change in precollege friendships” [29]) experienced by many students.

In our study, informed by friendsickness, maintaining existing relationships is one of the main drivers of using social media. Students actively interact with their friends from home by reading and responding to their posts. This may provide a safe opportunity for self-revelation to a more established connection in their network and a means of alleviating possible isolation.

Interestingly, although social media enables students to build new relationships, the visibility of their online activities raises the challenge of managing an online identity or presenting a single self to diverse audiences. The discrepancy between the “home” self and the “university” self acts as a strong barrier to posting personal material. Students may be connected to family members from whom they wish to conceal their new selves. This can even affect offline behaviors to avoid inappropriate photographs appearing [30].

Furthermore, university contacts are not a homogeneous group but may often include people from different cultures and backgrounds. This acts as an inhibitor because the increased diversity of the network leads to uncertain social norms and a higher potential for accidentally violating them. The risk of ostracism [31] or desire to avoid the disappointment of not receiving likes [32] leads students to avoid posting and sharing online. As a result, minimal interaction becomes common. Furthermore, when viewing profiles to evaluate new contacts, students realize that their profiles are viewed and judged by others in the same way. As they become conscious of their social media persona being judged, they edit it, not only to impress others but also to reflect and build their ideal selves.

For medical students, academic pressures from assignments and examinations drive them to use social media and support each other [5,33]. Online peer support in small groups is perceived as a low-risk environment by students. This can build genuinely supportive, reciprocal relationships, particularly in courses where a comprehensive set of skills is required, so that a student who is strong in one area may require support in another.

Importantly, by untangling and examining these factors, our findings show that academic use of social media and relationship management affect one another, which leads to stress and more conservative online behaviors. This prevents educators and publishers from promoting social media as a communication and sharing platform in education contexts.

Digital environments and social media in particular can enable interaction [34-36], collaboration [37], and information and resource sharing [38]. Social media has been advocated as an effective tool for medical education [4]. Our study suggests that it is necessary for educators to assist students to remove the identified inhibitors and to cope with their stress. For example, for students’ concerns about sharing learning materials with their peers on social media, platforms such as Facebook can offer services that enable students to access the shared learning material if they actively contribute to the academic discussion. This would encourage students to help each other rather than only receiving support from their peers. Open discussion and exchange of information would facilitate students’ learning and keep them updated with the knowledge advances in medicine.

### Limitations and Future Research

Several limitations of our study offer avenues for future research projects. First, to present a simple and parsimonious model, we considered stress as the main route to explain how inhibitors lead to conservative social media engagement and learning behaviors. It should be noticed that factors such as uncertain social norms might lead to cautious use of social media as well. For instance, gamification [39] or enhanced empowerment and hedonic well-being [40,41] as part of the online education platform design may strengthen user engagement and willingness to acquire critical new knowledge. Future research should explore other explanations and relationships among these factors to complement the framework proposed in this study.

Second, the research was limited by the relatively small number of participants. The research was qualitative and self-reported. It did not elicit information about how much time students spent on social media, or data to carry out a robust investigation into the nature of the content that they consumed and shared on various social media platforms. Future research should expand on the findings by collecting survey data or studying actual content shared on social media.

Third, this research took a single snapshot in the time of university adjustment. Future research should conduct a longitudinal study to explore medical students’ behavior change over time. For example, it would be intriguing to investigate whether the caution of using social media decreases by the end of university and increases again as students enter the workplace. In addition, in terms of supporting the student transition and learning outcomes, it would be interesting to track the relationship and situational pressures over time to see whether building certain kinds of personal relationships on social media during the early period translates into peer-to-peer academic support later on.

**Figure 3:**
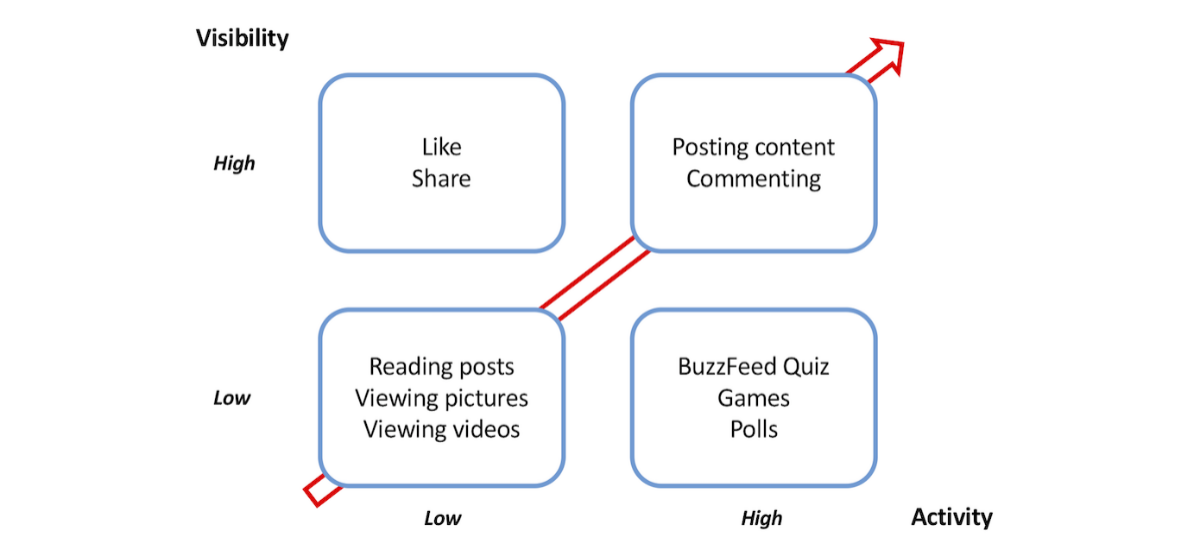
Effect of visibility and activity on user behaviors.

We also wonder whether enhanced transparency as part of online learning may reduce students’ stress and increase their willingness to engage in online learning tools. Prior work has shown that transparency of organizations is associated with greater levels of trust and people’s willingness to engage in open and responsible behaviors themselves when they see other relationship partners (eg, organizations) acting in a transparent way [42-45]. Furthermore, sharing critical information through customer education [45-48] has been demonstrated to positively affect people’s willingness to participate and offer critical and valuable feedback [49-51]. In addition to the factors identified in this study, how can social media be adapted effectively to reduce uncertainties [52,53] and to promote medical students’ learning and participation in the online learning environment? We invite future research to address this important question.

Finally, given student preferences, it seems that highly visual and seemingly low-risk platforms like Snapchat, with its disposable images, will continue to rise, and this should be a subject for additional research. There is empirical evidence for people getting instantaneously attached to digital services they use [54]. This can be because of an appealing interface, use of logos [55], the ethical standards of the service offering [56], or the versatility of the functional benefits provided [57]. Which social platform can medical students identify with based on the various benefits these platforms have to offer and their identification with work [58]? Interestingly, our interviews also revealed an increased preference by students to engage with Instagram rather than Facebook. Figure 3 highlights the effect of user visibility and activity on user behaviors, where the red arrow reflects increasing potential cost and therefore difficulty in driving the behavior. Additional research that tests the effects of visibility and activity on digital learning is rich in potential.

### Conclusion

This research explored student engagement with social media in the context of university adjustment. By presenting a view of students as an intentionally cautious group of social media users, we showed how students actively managed their engagement with social media as a significant part of their identity strategy. They carefully balanced the benefits of engagement, such as social support, peer learning support, and positive valence, with the potential risks to their identity. The more visible the engagement is, the more significance these engagement activities have in enhancing their identities. This has implications for understanding students’ online behaviors and the specific barriers that we should remove in order to use social media as a more effective learning tool.
